# Comparative chloroplast genomes of eleven *Schima* (Theaceae) species: Insights into DNA barcoding and phylogeny

**DOI:** 10.1371/journal.pone.0178026

**Published:** 2017-06-02

**Authors:** Xiang-Qin Yu, Bryan T. Drew, Jun-Bo Yang, Lian-Ming Gao, De-Zhu Li

**Affiliations:** 1 Germplasm Bank of Wild Species in Southwest China, Kunming Institute of Botany, Chinese Academy of Sciences, Kunming, Yunnan, China; 2 Key Laboratory for Plant Diversity and Biogeography of East Asia, Kunming Institute of Botany, Chinese Academy of Sciences, Kunming, Yunnan, China; 3 Department of Biology, University of Nebraska, Kearney, NE, United States of America; Saint Mary's University, CANADA

## Abstract

*Schima* is an ecologically and economically important woody genus in tea family (Theaceae). Unresolved species delimitations and phylogenetic relationships within *Schima* limit our understanding of the genus and hinder utilization of the genus for economic purposes. In the present study, we conducted comparative analysis among the complete chloroplast (cp) genomes of 11 *Schima* species. Our results indicate that *Schima* cp genomes possess a typical quadripartite structure, with conserved genomic structure and gene order. The size of the *Schima* cp genome is about 157 kilo base pairs (kb). They consistently encode 114 unique genes, including 80 protein-coding genes, 30 tRNAs, and 4 rRNAs, with 17 duplicated in the inverted repeat (IR). These cp genomes are highly conserved and do not show obvious expansion or contraction of the IR region. The percent variability of the 68 coding and 93 noncoding (>150 bp) fragments is consistently less than 3%. The seven most widely touted DNA barcode regions as well as one promising barcode candidate showed low sequence divergence. Eight mutational hotspots were identified from the 11 cp genomes. These hotspots may potentially be useful as specific DNA barcodes for species identification of *Schima*. The 58 cpSSR loci reported here are complementary to the microsatellite markers identified from the nuclear genome, and will be leveraged for further population-level studies. Phylogenetic relationships among the 11 *Schima* species were resolved with strong support based on the cp genome data set, which corresponds well with the species distribution pattern. The data presented here will serve as a foundation to facilitate species identification, DNA barcoding and phylogenetic reconstructions for future exploration of *Schima*.

## Introduction

The chloroplast (cp) is a type of plastid that is critical to the growth of most plants, playing a major role in photosynthesis and fixation of CO_2_ [1]. The cp genomes in angiosperms are circular DNA molecules with a highly conserved gene order and gene content, and range from 120 to 160 kb in length [2]. These genomes typically include two copies of an inverted repeat (IR) region that is separated by a large-single-copy (LSC) region and a small-single-copy (SSC) region [3]. Due to the rapid accumulation of genomic data gleaned from next-generation sequencing (NGS) technologies [4–6], more than 800 complete cp genomes of land plants have been sequenced (up to December 2016 from NCBI). The cp genome can provide valuable information for species identification, phylogeny and population genetic analyses [7–9]. It has also been postulated to be a potential ultra- or organelle-scale barcode for efficient plant species identification, especially for the taxonomically complex groups [10, 11].

*Schima*, with ca. 20 species, is an economically and ecologically important genus of the tea family (Theaceae). The genus is distributed in subtropical and tropical areas of East Asia, with 13 species (6 endemic) present in China [12]. Species of *Schima* are large trees and dominant elements of the subtropical evergreen broadleaved forests (SEBLFs) in East Asia [13, 14]. Some species are used as biological fire-resistant trees, and the wood is used for building and furniture [15, 16]. *Schima* is distinct from other genera within Theaceae, characterized by globose to oblate fruits and small reniform seeds with a marginal membranous wing. However, the infrageneric classification of *Schima* is complex and controversial due to a dearth of taxonomically diagnostic characters and high morphological similarity among species. This taxonomic uncertainty may hinder our exploitation and utilization of the genus.

Since its establishment as a genus, there has been much debate regarding the number of species within *Schima* [17]. Eighteen species were proposed in the second edition of the “Die Natürlichen Pflanzenfamilien” [18]. Bloembergen [19] regarded the genus as monotypic and subdivided *Schima wallichii* into nine geographically separated subspecies and three varieties. Airy-Shaw [20] recognized 15 species in *Schima*. Keng [21] accepted most of Bloembergen’s subspecies and raised them to the species level, and proposed that there were 10–15 species within the genus. The most recent treatment recognized ca. 20 species in *Schima* [12]. *Schima* is placed in tribe Gordonieae based on the results of molecular phylogenetic studies [22–24]. However, phylogenetic relationships within *Schima* are still unclear due to limited species sampling in previous studies, thus both species delimitations and phylogenetic reconstruction within *Schima* require further exploration.

Complete cp genomes have been shown to be effective in resolving interspecies phylogenetic relationships within *Camellia*, a genus in the sister tribe (Theeae) to Gordonieae [25, 26]. Here, we sequenced 11 cp genomes of the 13 Chinese *Schima* species. This study aims to: (1) investigate structural patterns of *Schima* cp genomes, (2) screen sequence divergence hotspots in the 11 *Schima* cp genomes, (3) explore simple sequence repeats (SSRs) among the 11 *Schima* cp genomes, (4) and reconstruct phylogenetic relationships among the 11 *Schima* species using the cp genome sequences. The results will provide abundant information for further studies regarding taxonomy, phylogeny, and population genetics of *Schima*, and will also assist in the exploration and utilization of the resources within the genus.

## Materials and methods

### Taxon sampling

In this study, we follow the classification of *Schima* from Min and Bartholomew [12]. Healthy and fresh leaves from 11 species of *Schima* were sampled from various localities across southern China (Table 1). Voucher specimens of each species were collected and deposited in the Herbarium of Kunming Institute of Botany, Chinese Academy of Sciences (KUN). *Gordonia lasianthus* and *Franklinia alatamaha* were used as outgroups in the phylogenetic analyses, and the cp genomes of these two species were obtained from our previous work (Yu et al., unpublished work).

**Table 1:** List of taxa sampled in this study, with the voucher, chloroplast genome size, Illumina reads and coverage depth information.

Taxon	Voucher specimen	Sources	Genome size	LSC length (bp)	SSC length (bp)	IR length (bp)	GC content (%)	No. reads (trimmed)	Mean coverage	GenBank No.
*Schima argentea*	YXQ041	Yunnan, China	157,245	87,222	18,091	25,966	37.43	7,448,533	620.1	KY406780
*Schima brevipedicellata*	YXQ069	Yunnan, China	157,227	87,202	18,089	25,968	37.44	687,249	1538.6	KY406758
*Schima crenata*	YXQ103	Hainan, China	157,288	87,232	18,104	25,976	37.44	701,233	1679.4	KY406755
*Schima khasiana*	YXQ070	Yunnan, China	157,252	87,208	18,112	25,966	37.43	459,809	1106.5	KY406794
*Schima multibracteata*	YXQ146	Guangxi, China	157,278	87,233	18,103	25,971	37.44	737,292	1882.5	KY406763
*Schima noronhae*	YXQ034	Yunnan, China	157,278	87,217	18,091	25,985	37.43	527,901	1284.3	KY406787
*Schima remotiserrata*	YXQ186	Hunan, China	157,284	87,229	18,103	25,976	37.43	562,801	1389.5	KY406749
*Schima sericans*	YXQ053	Yunnan, China	157,302	87,272	18,122	25,954	37.45	748,129	1735.2	KY406779
*Schima sinensis*	YXQ2902	Sichuan, China	157,297	87,243	18,102	25,976	37.45	10,152,425	716.8	KY406762
*Schima superba*	YXQ142	Guangxi, China	157,254	87,202	18,100	25,976	37.44	457,215	1221.9	KY406788
*Schima wallichii*	YXQ001	Yunnan, China	157,240	87,204	18,104	25,966	37.44	28,866	71.7	KY406795

Voucher specimens were deposited in the Herbarium of Kunming Institute of Botany (KUN), Chinese Academy of Sciences.

### DNA extraction, sequencing, chloroplast genome assembly

Total genomic DNA was isolated from fresh leaves (~100 mg) using the modified CTAB method (Doyle and Doyle 1987). Subsequently, the cp genomes were amplified using long-range PCR with fifteen primers [27]. The PCR products were fragmented for constructing short-insert (500 bp) libraries following the Illumina Nextera XT DNA library preparation instructions. Paired-end sequencing (250 bp) was performed on the Illumina MiSeq 2000 at the Laboratory of Molecular Biology of Germplasm Bank of Wild Species in Southwest China. Quality control of the raw sequence reads was performed using the NGS QC Tool Kit [28], with a cut-off value for percentage of read length and PHRED quality score as 80 and 30 following Yang et al. [5]. High-quality reads were assembled into contigs using the *de novo* assembler in CLC Genomics Workbench v6.5 (CLC Bio), using a *k*-mer of 64 and a minimum contig length of 500 base pairs (bp). The *de novo* contigs were assembled into complete chloroplast genomes followed the procedure of Yang et al. [5].

### Chloroplast genome annotation and comparisons

The complete cp genomes were annotated with the identification of introns and exons using DOGMA [29]. The positions of start and stop codons and boundaries between introns and exons were investigated according to the published cp genome of *Camellia taliensis* (NC022264). The annotated GenBank files were used to draw the circular chloroplast genome maps using OrganellarGenomeDRAW [30]. The mVISTA program [31] was employed in the LAGAN mode to detect the variation of the chloroplast genomes. The cp genome of *Schima sinensis* was used as a reference. Microsatellites (mono-, di-, tri-, tetra-, penta- and hexanucleotide repeats) were detected using Phobos v3.311 [32], with the parameters set to ten repeat units (≥ 10) for mononucleotide SSRs, six repeat units (≥ 6) for dinucleotide, four repeat units (≥ 4) for trinucleotide, four repeat units (≥ 4) for tetranucleotide, and three repeat units (≥ 3) for pentanucleotide and hexanucleotide SSRs. The percent variability for all protein-coding and noncoding (intergenic spacers and introns) regions of the cp genomes with an aligned length larger than 150 bp among the 11 *Schima* species was estimated in Geneious [33].

### Phylogenetic inference

The cp genomes were aligned using MAFFT v7.221 [34] under default settings (FFT-NS-2 strategy). One of the IRs was removed from the data set for the phylogenetic analysis. Poorly aligned regions (mainly introns and spacers) of the data set were realigned using the G-INS-i (accurate strategy) to improve the quality of the alignment. We used jModelTest v0.11 [35] to select the best-fitting nucleotide substitution models for maximum-likelihood (ML) according to the Akaike information criterion (AIC; Akaike, 1974). ML analysis was implemented in RAxML v8.20 [36]. We conducted a rapid bootstrap analysis (1000 replicates) and searched for the best-scoring ML tree simultaneously (the “-f a” option). Numbers of variable and informative sites were calculated in DnaSP v5.10 [37].

## Results

### Chloroplast genome features

**Fig 1:**
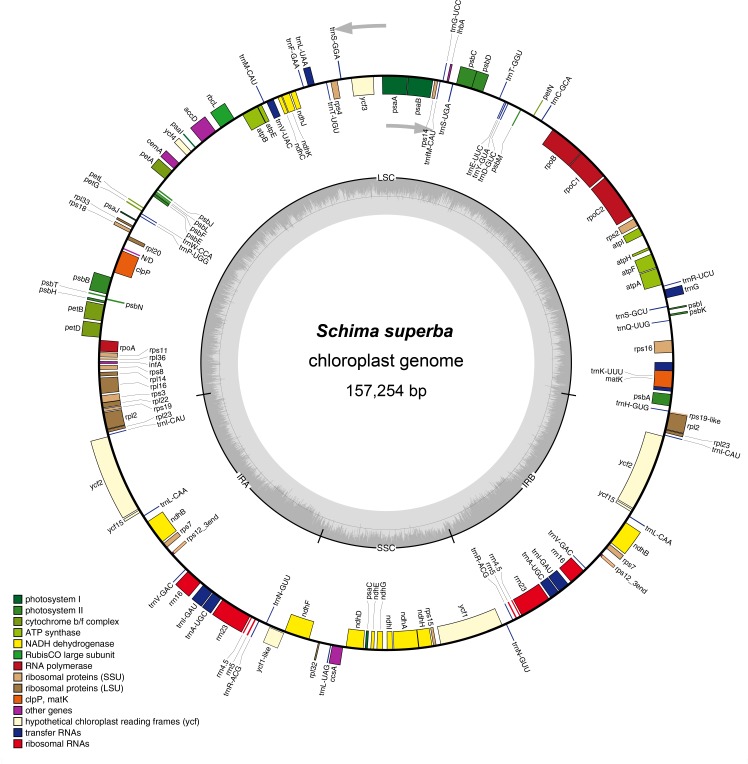
Gene map of *Schima superba* chloroplast genome.

**Fig 2:**
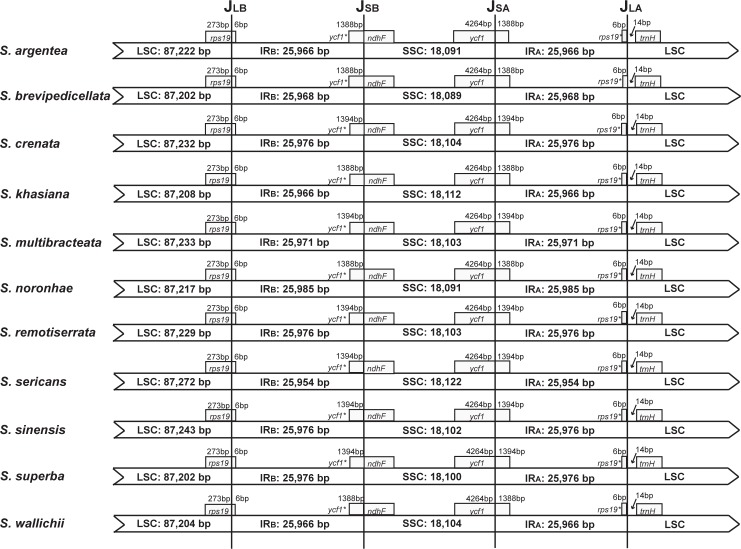
Comparisons of the border regions among the chloroplast genomes of 11 *Schima* species. ycf1^*^ (*ycf1*_like) and rps19^*^ (*rps19*_like) represent the incomplete duplication of the gene within the IR region.

Illumina paired-end sequencing of long-range PCR amplified cp DNA generated 28,866–10,152,425 clean reads for the 11 sampled *Schima* species, with mean coverage from 71.7 to 1882.5. The genome size ranged from 157,227 bp in *Schima brevipedicellata* to 157,302 bp in *Schima sericans* (Table 1). All of the 11 cp genomes showed typical quadripartite structure consisting of a pair of IR (25,954–25,985 bp) separated by the LSC (87,202–87,272 bp) and SSC (18,089–18,122 bp) regions (Table 1). The cp genome map of *Schima superba* is presented as a representative (Fig 1). Excluding the duplicated IR region, the 11 *Schima* cp genomes identically encoded 114 different genes that were arranged in the same order, including 80 protein-coding genes, 30 tRNAs and 4 rRNAs. Seventeen genes were duplicated in the IRs, with six protein-coding genes, four rRNA and seven tRNA genes. Twelve of the protein-coding genes and six of the tRNA genes contained introns. Fifteen out of those eighteen genes contained a single intron, while the other three (*clpP*, *rps12* and *ycf3*) had two introns. The 11 *Schima* cp genomes exhibited high similarity at the LSC/IR/SSC boundaries (Fig 2). The *rps19* gene crossed the LSC/IR_B_ (J_LB_) region with no variation of sequence length within the two parts. The SSC/IR_B_ (J_SB_) junction occurred between the *ycf1*_like (incompletely duplicated in IR_B_) and the 3’ end of *ndhF* gene, with the sequence length of *ycf1*_like gene within IR_B_ as 1388 or 1394. The *ycf1* gene crossed the SSC/IR_A_ (J_SA_) region, with 1388 or 1394 bp of *ycf1* within IR_A_. The *ycf1* related length changes were the only variation detected in these junctions. The LSC/IR_A_ (J_LA_) junction was located at the 3’ end of the *rps19*_like (6 bp; incompletely duplicated in IR_A_), with a 14 bp noncoding sequence between J_LA_ and *trnH* gene. In addition, we identified unusual start codons for four genes, ACG for *ndhD*, ATC for *psbI*, ATT for *psbT* and GTG for *rps19*.

### Chloroplast genome comparisons and divergence hotspots

**Fig 3:**
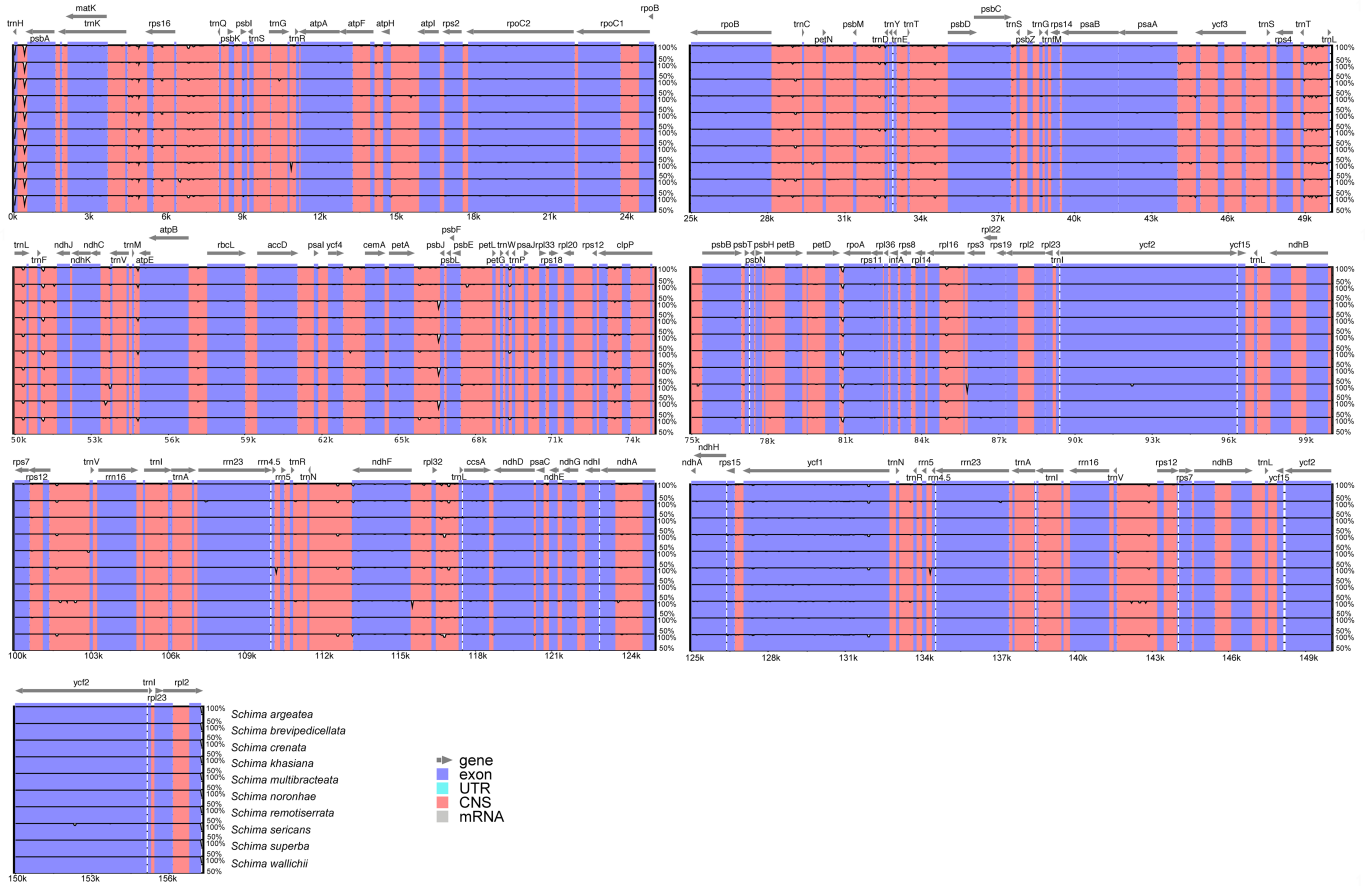
mVISTA percent identity plot comparison among the chloroplast genomes with *S*. *sinensis* as a reference.

**Fig 4:**
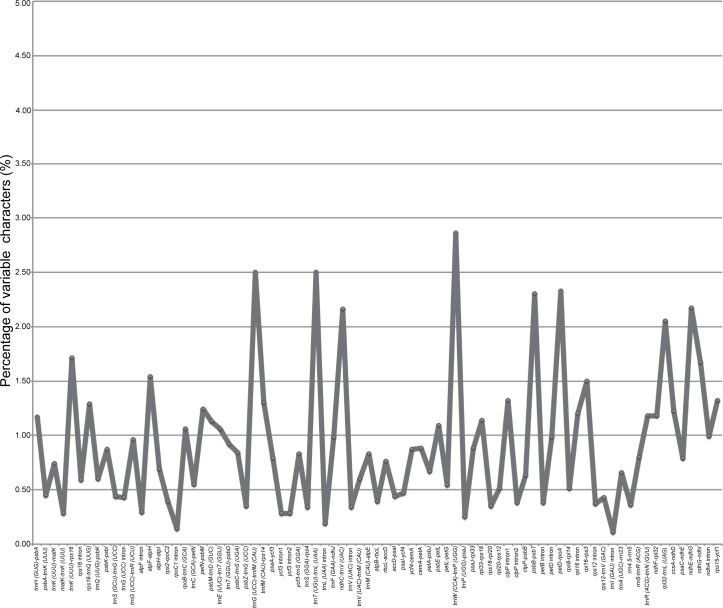
Percentage of variation in 79 variable noncoding regions of the 11 *Schima* chloroplast genomes. These regions are oriented according to their locations in the genome.

Sequence identity plots of the 11 *Schima* cp genomes, generated using mVISTA, are shown in Fig 3. The plots illustrate the high sequence similarity across the *Schima* cp genomes, with a sequence identity of 99.1%. Two (*ccsA* and *rps15*) of the 49 variable protein-coding (>150 bp) genes had a percentage of variation above 1.00% (Table 2), while 19 (>150 bp) had no variation. Both of the two core DNA barcodes (*rbcL* and *matK*) [38] showed extremely low sequence divergence (0.21% and 0.33%, respectively). Furthermore, the variation of *ycf1*, the proposed “most promising chloroplast DNA barcode” of land plants [39], was only 0.67%. Among the 79 noncoding (>150 bp) regions, the percentage of variation ranged from 0.11% to 2.85% (Fig 4 and Table 3). Fourteen fragments (*atpI-rps2*, *trnS* (*UGA*)*-psbZ*, *rps4-trnT* (*UGU*), *trnL* (*UAA*)*-trnF* (*GAA*), *petB-petD*, *rpl2* intron, *rpl23-trnI* (*CAU*), *ycf15-trnL* (*CAA*), *trnL* (*CAA*)*-ndhB*, *ndhB* intron, *trnV* (*GAC*)*-rrn16*, *rrn16-trnI* (*GAU*), *trnA* (*UGC*) intron, *trnN* (*GUU*)*-ndhF*) did not show any sequence variation. Eight potential mutational hotspots (*trnW* (*CCA*)*-trnP* (*UGG*), *trnT* (*UGU*)*-trnL* (*UAA*), *trnG* (*UCC*)*-trnfM* (*CAU*), *petD-rpoA*, *psbB-psbT*, *ndhE-ndhG*, *ndhC-trnV* (*UAC*), *rpl32-trnL* (*UAG*)) were identified, with the variation percentage exceeding 2.0% among the 11 sampled species (Fig 4 and Table 3). These eight highly variable hotspots may have the potential to be used as special DNA barcodes for identifying *Schima* species.

**Table 2:** Sequence divergence of 49 variable coding regions (>150 bp) from 11 chloroplast genomes of *Schima*, with one of the Inverted Repeat regions removed.

Fragments	Length (bp)	Aligned length (bp)	Variable positions	Nucleotide substitutions	Number of indels	Total length of indels	Percent variability (%)
*matK*	1527	1527	5	5	0	0	0.33
*psbK*	186	186	1	1	0	0	0.54
*psbI*	153–156	156	3	0	1	3	0.64
*atpA*	1524	1524	3	3	0	0	0.20
*atpF*	567	567	2	2	0	0	0.35
*atpI*	744	744	1	1	0	0	0.13
*rps2*	711	711	1	1	0	0	0.14
*rpoC2*	4137	4137	11	11	0	0	0.27
*rpoC1*	2061	2061	3	3	0	0	0.15
*rpoB*	3213	3213	11	11	0	0	0.34
*psbC*	1422	1422	3	3	0	0	0.21
*psaB*	2205	2205	2	2	0	0	0.09
*psaA*	2253	2253	6	6	0	0	0.27
*rps4*	606	606	2	2	0	0	0.33
*ndhK*	678	678	1	1	0	0	0.15
*ndhC*	363	363	2	2	0	0	0.55
*atpE*	402	402	1	1	0	0	0.25
*atpB*	1497	1497	1	1	0	0	0.07
*rbcL*	1428	1428	3	3	0	0	0.21
*accD*	1542	1542	4	4	0	0	0.26
*ycf4*	555	555	3	3	0	0	0.54
*cemA*	690	690	2	2	0	0	0.29
*petA*	963	963	2	2	0	0	0.21
*rpl20*	354	354	1	1	0	0	0.28
*rps12*	372	372	1	1	0	0	0.27
*clpP*	645	645	3	3	0	0	0.47
*psbB*	1527	1527	2	2	0	0	0.13
*petB*	663	663	4	4	0	0	0.60
*rpoA*	1014	1014	2	2	0	0	0.20
*rps11*	417	417	3	3	0	0	0.72
*infA*	234	234	1	1	0	0	0.43
*rps8*	408	408	1	1	0	0	0.25
*rpl14*	369	369	3	3	0	0	0.81
*rpl16*	411	411	2	2	0	0	0.49
*rps3*	657	657	2	2	0	0	0.30
*rpl22*	474	474	1	1	0	0	0.21
*ycf2*	6867–6873	6873	8	2	1	6	0.04
*rps7*	468	468	1	1	0	0	0.21
*ndhF*	2247–2253	2253	26	14	2	12	0.71
*rpl32*	162	162	1	1	0	0	0.62
*ccsA*	963	963	10	10	0	0	1.04
*ndhD*	1530	1530	3	3	0	0	0.20
*psaC*	246	246	1	1	0	0	0.41
*ndhE*	306	306	1	1	0	0	0.33
*ndhG*	531	531	3	3	0	0	0.56
*ndhA*	1092	1092	2	2	0	0	0.18
*ndhH*	1182	1182	2	2	0	0	0.17
*rps15*	273	273	3	3	0	0	1.10
*ycf1*	5652–5658	5658	43	37	1	6	0.67

**Table 3:** Sequence divergence of 79 variable noncoding loci (>150 bp) from 11 chloroplast genomes of *Schima*, with one of the invert repeat regions removed.

Fragments	Length (bp)	Aligned length (bp)	Variable positions	Nucleotide substitutions	Number of indels	Total length of indels	Percent variability (%)
*trnH (GUG)-psbA*	395–426	428	37	4	1	33	1.17
*psbA-trnK (UUU)*	220	220	1	1	0	0	0.45
*trnK (UUU)-matK*	270	270	2	2	0	0	0.74
*matK-trnK (UUU)*	712–713	713	2	1	1	1	0.28
*trnK (UUU)-rps16*	809–818	819	22	10	4	12	1.71
*rps16* intron	838–844	845	10	3	2	7	0.59
*rps16-trnQ (UUG)*	1689–1698	1700	36	18	4	18	1.29
*trnQ (UUG)-psbK*	333	333	2	2	0	0	0.60
*psbK-psbI*	345	345	3	3	0	0	0.87
*trnS (GCU)-trnG (UCC)*	681–682	682	3	3	0	0	0.44
*trnG (UCC)* intron	690–696	696	8	2	1	6	0.43
*trnG (UCC)-trnR (UCU)*	276–311	311	37	2	1	35	0.96
*atpF* intron	701	701	2	2	0	0	0.29
*atpF-atpH*	387–389	389	7	5	1	2	1.54
*atpH-atpI*	1143–1153	1153	15	4	4	11	0.69
*rps2-rpoC2*	254–255	255	1	0	1	1	0.39
*rpoC1* intron	732–734	734	2	0	1	2	0.14
*rpoB-trnC (GCA)*	1211–1222	1222	26	9	4	17	1.06
*trnC (GCA)-petN*	722–727	727	8	3	1	5	0.55
*petN-psbM*	1123–1125	1125	15	13	1	2	1.24
*psbM-trnD (GUC)*	1136–1153	1153	27	8	5	19	1.13
*trnE (UUC)-trnT (GGU)*	473	473	5	5	0	0	1.06
*trnT (GGU)-psbD*	1513–1517	1519	19	11	3	8	0.92
*psbC-trnS (UGA)*	234–239	239	6	1	1	5	0.84
*psbZ-trnG (UCC)*	283	283	1	1	0	0	0.35
*trnG (UCC)-trnfM (CAU)*	157–159	160	5	2	2	3	2.50
*trnfM (CAU)-rps14*	154	154	2	2	0	0	1.30
*psaA-ycf3*	747–755	755	17	3	3	14	0.79
*ycf3* intron1	721	721	2	2	0	0	0.28
*ycf3* intron2	726	726	2	2	0	0	0.28
*ycf3-trnS (GGA)*	839–842	842	8	4	3	4	0.83
*trnS (GGA)-rps4*	293	293	1	1	0	0	0.34
*trnT (UGU)-trnL (UAA)*	982–999	999	46	19	6	27	2.50
*trnL (UAA)* intron	522–529	529	7	0	1	7	0.19
*trnF (GAA)-ndhJ*	704–714	715	21	3	4	18	0.98
*ndhC-trnV (UAC)*	400–414	417	36	3	6	33	2.16
*trnV (UAC)* intron	585	585	2	2	0	0	0.34
*trnV (UAC)-trnM (CAU)*	166	166	1	1	0	0	0.60
*trnM (CAU)-atpE*	229–238	240	13	0	2	13	0.83
*atpB-rbcL*	765–768	768	5	2	1	3	0.39
*rbcL-accD*	525–526	526	4	3	1	1	0.76
*accD-psaI*	681–683	683	4	2	1	2	0.44
*psaI-ycf4*	423–425	425	3	1	1	2	0.47
*ycf4-cemA*	909–915	915	13	7	1	6	0.87
*cemA-petA*	220–228	228	8	0	2	8	0.88
*petA-psbJ*	1035–1042	1043	12	4	3	8	0.67
*psbE-petL*	1277–1287	1287	22	12	2	10	1.09
*petL-petG*	185–186	186	1	0	1	1	0.54
*trnW (CCA)-trnP (UGG)*	170–175	175	9	4	1	5	2.86
*trnP (UGG)-psaJ*	391–393	393	2	0	1	2	0.25
*psaJ-rpl33*	455–457	457	5	3	1	2	0.88
*rpl33-rps18*	175–176	176	2	1	1	1	1.14
*rps18-rpl20*	284	284	1	1	0	0	0.35
*rpl20-rps12*	786	786	4	4	0	0	0.51
*clpP* intron1	598–605	607	13	3	5	10	1.32
*clpP* intron2	797–798	798	3	2	1	1	0.38
*clpP-psbB*	473–479	479	8	2	1	6	0.63
*psbB-psbT*	172–174	174	5	3	1	2	2.30
*petB* intron	787	787	3	3	0	0	0.38
*petD* intron	711	711	7	7	0	0	0.98
*petD-rpoA*	200–213	215	15	0	5	15	2.33
*rps8-rpl14*	196	196	1	1	0	0	0.51
*rpl16* intron	996–998	998	13	11	1	2	1.20
*rpl16-rps3*	150–200	200	52	2	1	50	1.50
*rps12* intron	536	536	2	2	0	0	0.37
*rps12-trnV (GAC)*	1602–1619	1619	29	2	5	27	0.43
*trnI (GAU)* intron	938	938	1	1	0	0	0.11
*trnA (UGC)-rrn23*	152	152	1	1	0	0	0.66
*rrn4*.*5-rrn5*	256–275	275	19	0	1	19	0.36
*rrn5-trnR (ACG)*	248–249	249	2	1	1	1	0.80
*trnR (ACG)-trnN (GUU)*	595	595	7	7	0	0	1.18
*ndhF-rpl32*	825–847	851	32	6	4	26	1.18
*rpl32-trnL (UAG)*	908–926	928	36	13	6	23	2.05
*ccsA-ndhD*	240–244	245	6	1	2	5	1.22
*psaC-ndhE*	251–254	254	4	1	1	3	0.79
*ndhE-ndhG*	230	230	5	5	0	0	2.17
*ndhG-ndhI*	359–360	360	6	5	1	1	1.67
*ndhA* intron	1106–1111	1112	16	8	3	8	0.99
*rps15-ycf1*	379	379	5	5	0	0	1.32

The aligned length of the complete cp genome (with one of the IR removed) among the 11 *Schima* species was 130,508 bp, with the total number of variable and parsimony informative (PI) sites being 1,121 bp and 261 bp, respectively. This data set contained 131 indels with a total length of 586 bp, and the percent variability was 0.51%. These results indicate that the global variation of the cp genome within *Schima* is extremely low. A similar pattern was reported in other long-lived plants [40–42]. The ability to identify species within the genus using cp genome data needs to be assessed by sampling multiple individuals per species, even though the phylogenetic analyses have most of the species separated from each other (see below).

### SSR polymorphisms

In total, 58 cpSSRs, including 55 mononucleotide (A, T), 1 dinucleotide (AT) and 2 trinucleotide (ATT, TTA) repeats were detected within the 11 *Schima* cp genomes. No tetranucleotide, pentanucleotide or hexanucleotide repeats were observed. The mononucleotide repeat (A, T) was found to be the most abundant, with repeat numbers of 10, 11 and 12 (Table 4). The proportion of A and T repeats in mononucleotide repeat unit was 43.64% and 56.36%, respectively. Only one SSR locus with a different repeat unit (C) was detected in the *trnG (UCC)-trnfM (CAU)* intergenic spacer region. Within the 11 *Schima* cp genomes, SSR loci were primarily located in the LSC region (89.09%), followed by the SSC portion (14.55%), with only one present in the IR region (*rrn5-trnR* (*ACG*)) (Table 4). One SSR locus was detected in the protein-coding gene *psbI*, with all others located in gene spacers and introns. No SSRs were found in the tRNAs and rRNAs. The mononucleotide repeat (A) in *trnH-psbA* was the most variable SSR, with the size ranging from 12 to 42 bp. The cpSSRs of the 11 *Schima* species represented here showed abundant variation, and could be useful for research at the population level. They will provide complementary data to the SSR markers of *Schima* identified from the nuclear genome [43].

**Table 4:** Location of SSR loci within the 11 *Schima* genomes.

No.	Motif	Location	Region	Repeat length
1	A	*trnH-psbA*	LSC	12–43
2	A	*trnK (UUU)* intron	LSC	10–11
3	A	*trnK (UUU)-rps16*	LSC	9–10
4	A	*trnK (UUU)-rps16*	LSC	11–14
5	A	*trnK (UUU)-rps16*	LSC	8–10
6	T	*rps16-trnQ (UUG)*	LSC	9–14
7	A	*rps16-trnQ (UUG)*	LSC	8–10
8	T	*rps16-trnQ (UUG)*	LSC	8–11
9	T	*psbI*	LSC	10, 13
10	A	*atpA-atpF*	LSC	14,15
11	T	*atpF-atpH*	LSC	9–12
12	AT	*atpF-atpH*	LSC	12,14
13	A	*atpH-atpI*	LSC	13–20
14	T	*atpH-atpI*	LSC	12,13
15	A	*rps2-rpoC2*	LSC	10,11
16	A	*rpoC2-trnC (GCA)*	LSC	9,10
17	T	*psbM-trnD (GUC)*	LSC	10,11
18	T	*trnT (GGU)-psbD*	LSC	9,10
19	A	*trnT (GGU)-psbD*	LSC	9–14
20	T	*psbC-trnS (UGA)*	LSC	11–16
21	C	*trnG (UCC)-trnfM (CAU)*	LSC	8, 10
22	A	*trnG (UCC)-trnfM (CAU)*	LSC	9,10
23	A	*psaA-ycf3*	LSC	10–14
24	A	*ycf3-trnS (GGA)*	LSC	11,12
25	A	*ycf3-trnS (GGA)*	LSC	11,12
26	A	*trnT (UGU)-trnL (UAA)*	LSC	13,14
27	T	*trnF (GAA)-ndhJ*	LSC	9, 10
28	T	*ndhC-trnV (UAC)*	LSC	8–16
29	T	*ndhC-trnV (UAC)*	LSC	9–12
30	TTA	*ndhC-trnV (UAC)*	LSC	3,12
31	T	*ndhC-trnV (UAC)*	LSC	9,10
32	T	*trnM (CAU)-atpE*	LSC	9–12
33	T	*atpB-rbcL*	LSC	12–15
34	T	*rbcL-accD*	LSC	11,12
35	T	*accD-psaI*	LSC	13–15
36	T	*psaI-ycf4*	LSC	10–12
37	T	*petA-psbJ*	LSC	10–13
38	T	*petA-psbJ*	LSC	10,11
39	T	*petL-petG*	LSC	10,11
40	T	*trnP (UGG)-psaJ*	LSC	10–12
41	T	*rpl20-rps12*	LSC	6,10
42	T	*clpP* intron	LSC	10,11
43	A	*clpP* intron	LSC	9–11
44	A	*clpP* intron	LSC	9,10
45	ATT	*clpP-psbB*	LSC	6,12
46	A	*psbB-psbT*	LSC	8–10
47	T	*petD-rpoA*	LSC	9,10
48	A	*petD-rpoA*	LSC	9–11
49	A	*petD-rpoA*	LSC	9,10
50	A	*rrn5-trnR (ACG)*	IR	9,10
51	T	*ndhF-rpl32*	SSC	9–12
52	T	*ndhF-rpl32*	SSC	10,11
53	A	*rpl32-trnL (UAG)*	SSC	10,11
54	T	*rpl32-trnL (UAG)*	SSC	10–13
55	T	*ccsA-ndhD*	SSC	10–13
56	T	*psaC-ndhE*	SSC	9–12
57	T	*ndhG-ndhI*	SSC	10,11
58	A	*ndhA* intron	SSC	9,10

### Phylogenetic analyses

**Fig 5:**
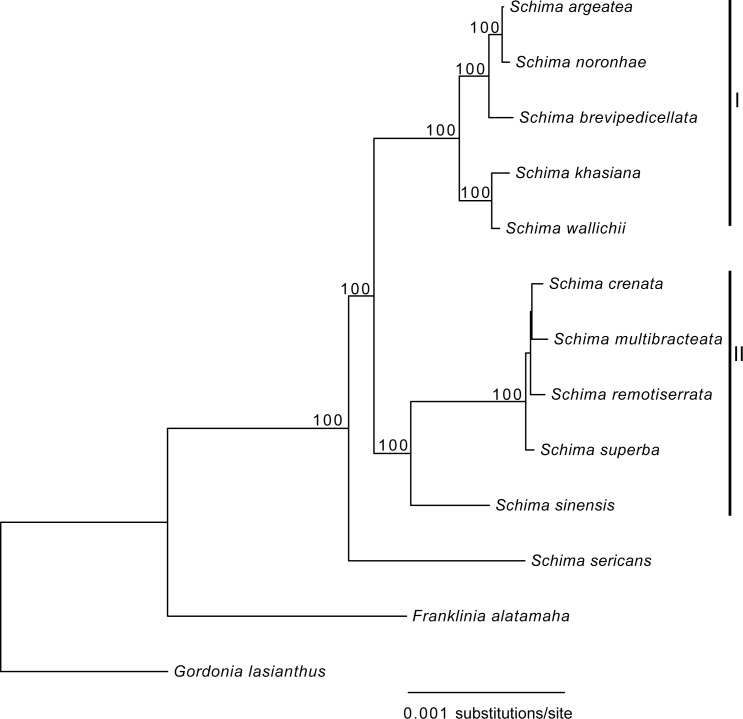
Phylogenetic relationships among the 11 *Schima* species. The phylogenetic tree was reconstructed using the whole chloroplast genome data set minus a copy of the IR region. Numbers above the branches show bootstrap support values that are above 80%.

The data matrix we used for phylogenetic estimation consisted of an alignment containing entire cp genomes with one of the IRs removed. This data set was comprised of 131,113 nucleotide positions, with 2,508 variable sites (1.91%) and 427 PI sites (0.33%). ML analysis resulted in a well-resolved tree, with eight of the 10 nodes supported by 100% bootstrap values (BS). All *Schima* species grouped into a strongly supported clade (BS = 100%, Fig 5), indicating *Schima* is monophyletic. Two main clades were recovered, with *Schima sericans* being sister to those two lineages. Five species (*S*. *argentea*, *S*. *brevipedicellata*, *S*. *khasiana*, *S*. *noronhae*, *S*. *wallichii*) formed clade I (BS = 100%, Fig 5). The remaining five species (*S*. *sinensis*, *S*. *superba*, *S*. *remotiserrata*, *S*. *multibracteata* and *S*. *crenata*) grouped in clade II (BS = 100%, Fig 5). The branch leading to *S*. *superba* and three closely related species is extremely short, and bootstrap support values for two internal nodes within this clade are less than 80%.

## Discussion

### Chloroplast genome features and comparison within Theaceae

Prior to this study, *Camellia* was the only genus within Theaceae to have its cp genome sequenced [25, 26]. In the present study, we sequenced cp genomes of 11 species from *Schima*. The cp genomes all displayed typical quadripartite structure (Fig 1), which is consistent amongst most lineages of angiosperms [2]. The expansion and contraction of the IR region is considered to be the primary mechanism affecting length variation of angiosperm cp genomes, as demonstrated in Trochodendraceae [44] and Apiales [45]. However, only minor variation was detected at the SSC/IR_A_ boundary of all of the 11 *Schima* cp genomes (Fig 2). Although the genes located at the IR junctions are identical in cp genomes of *Schima* and *Camellia*, the overall cp genome sequences of *Schima* are more homogenous as compared to *Camellia*, which was suggested to show more differences at the junction regions [26]. The cp genomes of *Schima* encode the same set of protein-coding genes as previously reported *Camellia* species, with the exception of *Orf 42* and *Orf 188* which were reported in *Camellia* [25], but not in other Ericales members such as *Actinidia* (Actinidiaceae) and *Ardisia* (Primulaceae) [46, 47]. For the whole cp genomes of *Schima*, 37 tRNA genes were annotated, which is consistent with Huang et al. [26]. However, 38 tRNA genes were found in Yang et al. [25], due to a redundant annotation of *trnP (UGG)* in their study. As compared with sequences of *Camellia*, no significant structural rearrangements such as inversions or changes of gene locations were found in the 11 *Schima* cp genomes. The high sequence similarity across the *Schima* cp genomes (Fig 3) may be associated with long generation time and recent radiation.

### Potentially specific DNA barcodes for *Schima*

Since the concept of DNA barcoding was proposed over a decade ago [48], substantial efforts have been made to develop DNA barcodes possessing both high universality and efficiency. Kress et al. [49] suggested that the internal transcribed spacer (ITS) and *trnH-psbA* spacer region had potential as useful DNA barcode regions for flowering plants. Hollingsworth et al. [38] later advocated *matK* and *rbcL* as a two-locus core barcode for land plants after comparing seven leading candidate loci, subsequently the nrDNA ITS was recommended to be incorporated into core barcode based on a large-scale sapmpling of seed plants [50]. Dong et al. [39] proposed that *ycf1* was the most variable loci of the cp genome, which might be a promising DNA barcode performing better than existing plastid candidate barcodes of land plants. However, all of the five candidate protein-coding DNA barcodes (*matK*, *rbcL*, *rpoB*, *rpoC1* and *ycf1*) showed extremely low sequence variation (<1.00%), and the other three fragments are also not among the most variable spacers. The eight potential mutational hotspots (*trnW* (*CCA*)*-trnP* (*UGG*), *trnT* (*UGU*)*-trnL* (*UAA*), *trnG* (*UCC*)*-trnfM* (*CAU*), *petD-rpoA*, *psbB-psbT*, *ndhE-ndhG*, *ndhC-trnV* (*UAC*), *rpl32-trnL* (*UAG*)) (Fig 4 and Table 3) identified in this study could be suitable barcodes for *Schima*. Recently, using the cp genome as a possible ultra- or organelle-scale barcode for efficient plant species identification was discussed [10, 11]. The high phylogenetic resolution among closely related species of *Schima* (Fig 5) suggests that the cp genome may indeed be useful as an organelle-scale barcode for species identification of *Schima*. Further studies based on sampling at the population scale are needed to evaluate the efficiency of the barcodes mentioned above and also the cp genome as an organelle-scale barcode.

### Phylogenetic relationships among species of *Schima*

The cp genome has been suggested to be useful for phylogenetic reconstructions at low taxonomic levels [7, 8, 10, 51]. Interspecies phylogenetic relationships within *Camellia* (Theaceae) were well-resolved using cp genome data [25, 26]. In the present study, based on a recent classification of the genus [12], 11 out of 13 *Schima* species occurring in China were represented. The phylogenetic relationships within *Schima* were well resolved with strong support based on cp genome sequences (Fig 5). Therefore, our study indicates that the complete cp genome has significant potential to resolve the low level phylogenetic relationships. *Schima sericans*, the first diverging lineage among sampled species, is distributed in southeastern Xizang and northwestern Yunnan in China. *Schima sericans* was sister to the remaining taxa, which formed two clades. Clade I includes five species (*S*. *argentea*, *S*. *brevipedicellata*, *S*. *khasiana*, *S*. *noronhae* and *S*. *wallichii*) that are primarily distributed in southwestern China and Indochina. Clade II comprises five species (*S*. *sinensis*, *S*. *superba*, *S*. *remotiserrata*, *S*. *multibracteata* and *S*. *crenata*) that mainly occur within central and eastern China (Fig 5). The phylogenetic relationships within *Schima* found here correspond well with the geographic distribution pattern, but do not match well with morphology. *Schima* was classified into two groups based on the shape of the leaf margin (entire or serrate) [12]. However, all of the species within clade I possess a serrate leaf margin except *S*. *khasiana*. Likewise, *S*. *multibracteata* is the only species with an entire leaf margin in clade II (Fig 5). Our results suggest that the taxonomic value of leaf margin shape should be reassessed for classification of *Schima*. Additionally, the branch length of the clade including *S*. *superba* and three closely related species is extremely short, indicating that these four species have recently diversified, or perhaps illustrating past hybridization within the group. These results indicate that the current treatment of the genus needs to be reevaluated by integrating more types of evidence.
